# Homocysteine: Canary in the Coal Mine or Hidden Threat? A Biochemical Study on the Role of Plasma Thiols

**DOI:** 10.1096/fj.202500677RR

**Published:** 2025-06-27

**Authors:** Daniela Giustarini, Sante Colella, Isabella Dalle‐Donne, Ranieri Rossi

**Affiliations:** ^1^ Department of Biotechnology, Chemistry and Pharmacy University of Siena Siena Italy; ^2^ Center for Colloid and Surface Science (CSGI), University of Florence Florence Italy; ^3^ Department of Biosciences University of Milan Milan Italy

**Keywords:** cardiovascular diseases, glutathione, homocysteine, plasma thiols, thiol to disulfide exchange reactions

## Abstract

Homocysteinemia is routinely measured as a biomarker of cardiovascular risk, but its pathogenic role remains controversial because it is unclear whether—and how—interventions to lower homocysteine levels provide real benefit. In the present original study, we analyzed in detail the effects of oxidative stress, thiol‐disulfide exchange reactions, and plasma thiol levels on homocysteinemia. We conducted a clinical study in a group of healthy, homogeneous individuals (*n* = 62) in which the different redox forms of plasma thiols and several biomarkers of oxidative stress were determined. Homocysteine was characterized by the fact that it was almost completely present as mixed protein disulfide (about 80%–85%). A strong inverse correlation was found between total homocysteine and glutathione concentrations, whereas no correlation was found between homocysteine and oxidative stress markers. The observation that oxidative stress does not affect total homocysteine levels in plasma was confirmed by in vitro treatments of human blood with a special device that allows slow delivery of oxidants. Experiments with cultured cells showed that they can release glutathione in large quantities with different kinetics over time. In addition, a strong inverse correlation between GSH and total homocysteine has been demonstrated in the plasma of humans of different ages and in mammalian species. All these data support the hypothesis that GSH, once released from cells, can trigger a series of thiol‐disulfide exchange reactions leading to the cleavage of protein‐bound homocysteine and the increase of free homocysteine, thus promoting its excretion. It can therefore be concluded that homocysteinemia can be regulated by the release of GSH from cells and that, consequently, total homocysteine in plasma can be considered a biomarker of cardiovascular risk without necessarily having a direct causal role. The specificity of this process must be taken into account when investigating the pathogenetic role of homocysteine.

## Graphical abstract

The figure illustrates how high plasma glutathione levels can promote the dethiolation of homocysteinylated proteins, leading to the release of homocysteine. The freed homocysteine is then either excreted through the kidneys or further processed via cellular metabolism, supporting homocysteine clearance and metabolic balance.

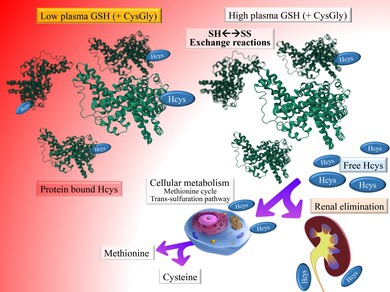

## 1 Introduction

Significantly elevated homocysteine levels in human plasma and urine were first described in certain rare inborn errors of metabolism known as homocystinuria. This condition is due to a recessive disorder of homocysteine metabolism involving the enzymes cystathionine‐β‐synthase (CBS) or methylenetetrahydrofolate reductase (MTHFR) [1]. The elevated homocysteine levels in homozygous individuals (up to 500 μM) impair collagen cross‐linking, leading to lens slippage, osteoporosis, scoliosis, a long, thin body and a high arched palate. Elevated homocysteine levels also damage the vascular epithelium and lead to the formation of thrombi, which are the main cause of death [2].

More than 50 years ago, McCully first reported that even moderately elevated blood homocysteine levels could be a possible cause of vascular abnormalities and thrombosis in individuals without homocystinuria. In short, the hypothesis was that, given that untreated young homocystinuria patients develop aggressive vascular disease, even very moderately elevated homocysteine could itself be a risk factor or amplify the effects of other risk factors to cause early‐onset vascular disease [3]. This condition is more common and may be due to several factors: (i) heterozygous defects for CBS or MTHFR genes; (ii) dietary causes: homocysteine may increase in individuals without genetic disorders due to a deficiency of one or more of the three key nutrients folate, cobalamin or vitamin B6 as a result of diet, malabsorption or other as yet undefined causes. Some of these individuals who appear to be normal with respect to the metabolism of the three nutrients may have underlying, as yet unidentified genetic variants; (iii) clinical conditions in which the metabolism of homocysteine is impaired, particularly renal disease [4]. Uremic patients have elevated plasma homocysteine levels [5], as do renal transplant recipients with mildly impaired renal function.

The normal values for homocysteine in the serum of healthy, fasting people are between 5 and 15 μM. Patients who exceed this value are classified as hyperomocysteinemic and divided into three categories: mild (15–30 μM), moderate (30–100 μM), severe (> 100 μM). According to various studies, the prevalence of mild, moderate and severe hyperomocysteinemia is 5%–10%, 0.5%–1% and 0.01%–0.03% respectively [6]. According to Humphrey et al. [7], every 5 μM rise in serum concentration corresponds to a 20%–30% increase in the risk of ischemic heart disease. Based on these considerations and since a considerable percentage of the population can be counted among the groups with increased cardiovascular risk, a wealth of literature was written in the following decades with the aim of investigating the epidemiology of the phenomenon and the pathophysiological causes as well as the biochemical nature.

Dozens of observational studies have shown that a homocysteine level above 15 μM is a risk factor for cardiovascular events (as summarized in some meta‐analyzes [8, 9]). In addition, numerous experimental studies in animals have shown that hyperhomocysteinemia can cause damage to blood vessel walls, pro‐inflammatory effects, oxidative stress, and endothelial dysfunction [10]. Several pathways by which homocysteine levels can damage endothelial cells and lead to atherosclerosis have been described in detail [11, 12, 13]. It is thought to have a direct pathogenic effect by causing oxidative modification of lipoproteins and subsequent precipitation of aggregates. It may also generate reactive oxygen species and impair basal production of nitric oxide, thereby preventing vasodilatation. Furthermore, in vitro studies have shown that homocysteine increases the production of various pro‐inflammatory cytokines such as monocyte chemoattractant protein 1 or interleukin‐8, a T‐lymphocyte and neutrophil chemoattractant, thereby promoting the development and progression of atherosclerosis [14]. However, treatment with homocysteine‐lowering vitamins (folic acid and vitamin B_12_) did not reduce the risk of recurrent cardiovascular disease after acute myocardial infarction [15] or the risk of major cardiovascular events in patients with vascular disease [16], nor did it reduce the incidence of ischemic stroke, although it did result in a significant reduction in circulating homocysteine (~30%) [17]. Currently, there is only weak (and controversial) evidence for a reduction in cardiovascular risk with homocysteine‐lowering interventions [18, 19], and homocysteine is mostly not considered a causal risk factor for cardiovascular disease, which is confirmed by both the American College of Clinical Cardiology and the European guidelines on cardiovascular disease prevention in clinical practice. However, the debate remains, especially after a recent Cochrane review found a small difference in favor of homocysteine‐lowering interventions compared to placebo in relation to strokes [20].

One of the weaknesses of these observational studies linking hyperhomocysteinemia to cardiovascular disease is that the molecular pathogenesis process has not been adequately explained. Although animal models suggest a link between mild hyperhomocysteinemia and cardiovascular disease, there is still no confirmation of both the biochemical origin of cell damage and evidence that therapy that can lower homocysteine levels will in turn reduce the risk of cardiovascular events.

In the present study, we first attempted to determine plasma homocysteine levels in a carefully selected group of subjects, eliminating or reducing confounding factors as much as possible. In particular, all samples were taken over a 2‐month period (May–June) to limit the influence of seasonal fluctuations in the availability of fruit and vegetables. The age range of the subjects was limited to 20 years, as the influence of age on homocysteinemia is well known [6], and selection was based on diet, ethanol consumption and various other factors. Based on the observation that an increase in plasmatic low molecular weight thiol is associated with a decrease in circulating homocysteine, we hypothesized that homocysteine might be an indicator that something is going wrong, a kind of “canary in the coal mine” that is itself a dangerous molecule for the cardiovascular system. The analytical method used for homocysteine measurement was the “gold standard” (i.e., HPLC), and the various redox forms of the major thiols in plasma were measured after stabilization with a proper solution added to whole blood immediately after collection [21].

## 2 Methods

### 2.1 Materials and Study Group

Citrate buffer solution pH 4.3 (thiol stabilizing solution) was prepared by mixing 0.5 M sodium citrate with 0.5 M citric acid and stored at −20°C. All other reagents were purchased from Merck (Milan, Italy) unless otherwise stated. The equipment used for the in vitro experiments on oxidant‐treated blood was purchased from LabOmak (Siena, Italy).

Analytical analyses were performed using an Agilent 1100 HPLC with fluorometric detector (Agilent Technologies, Milan, Italy) equipped with a Zorbax Eclipse XDB‐C18 4.6 × 150 mm, 5 μm column (Agilent Technologies) and a UV–Vis spectrophotometer (Jasco, V‐750).

The study group consisted of 62 consenting volunteers (32 women and 30 men, age range 31–49 years) who were recruited from among coworkers, relatives, and friends and verbally consented to the study (Table 1). None of the participants were active smokers and all were abstinent or drank less than 20 g of alcohol per week (equivalent to a medium glass of wine or a pint of beer). All participants reported being in good health and none of them had any abnormalities on physical examination or routine blood and urine laboratory tests performed no more than 6 months previously (routine blood chemistry including lipid profile, blood glucose, complete blood count, comprehensive metabolic panel, kidney, liver and heart functions, uric acid, electrolytes, urinary iron and glucose). All subjects received a free diet and reported regular moderate physical activity (e.g., walking, cycling or light exercise without intensive training) and regular sleep, with an average sleep duration of almost 8 h per night.

**TABLE 1:** Clinical characteristics of the subjects considered for the study. In brackets the range interval is reported. *n* = 62.

	Value
Characteristics	
M/F	30/32
Age	40.3 ± 6.5 (31–49)
Males weight (kg)	73.2 ± 5.1 (69–84)
Females weight (kg)	62.9 ± 5.6 (53–76)
Males height (cm)	176 ± 7.0 (168–191)
Females height (cm)	169 ± 10 (156–177)
Male BMI (kg/m^2^)	23.5 ± 1.9 (21.4–24.8)
Female BMI (kg/m^2^)	22.1 ± 2.8 (19.9–24.3)
Food daily intake	
Energy (kcal/day) M	2110 ± 136
Energy (kcal/day) F	2086 ± 174
Protein (g/day) M	84.7 ± 10.2
Protein (g/day) F	80.5 ± 8.85
Fat (g/day) F	53 ± 6
Carbohydrate (g/day) M	263 ± 15
Carbohydrate (g/day) F	251 ± 23

The evaluation of the diet showed that none of the test subjects had habits that could impair homocysteine metabolism (e.g., folic acid deficiency). The intake of N‐acetylcysteine, vitamins C and E as well as NSAIDs was avoided at least in the 2 months prior to the analyses. The basal homocysteine level was not considered an exclusion criterion.

### 2.2 Nutrition Study Group

A semi‐quantitative questionnaire with 360 items (both frequency and portion size) on frequency of food consumption [22] was completed by a registered dietitian. Frequency of consumption was divided into nine categories: never or rarely, once a month, two to three times a month, once or twice a week, three to four times a week, five to six times a week, once a day, twice a day, or three or more times a day. In order to minimize seasonal variations due to differences in diet, all samples were taken in the months of May–June. The dietary profile was assessed by a registered dietitian and subjects were included in the study if they were in the middle quartiles of the Food Frequency Questionnaire in terms of protein, fat, carbohydrate, and saturated/unsaturated fat intake. The assessment of nutritional status also included measurements of body mass (body mass index, BMI) and muscle mass (anthropometric indices of the arms) [23].

### 2.3 Processing of the Samples

Blood samples (3 mL) were collected from the antecubital vein in the morning after fasting for approximately 12 h in evacuated plastic tubes containing K_3_EDTA and 0.01 mM acivicin (final concentration). The sample was divided into different aliquots for analysis of thiols and disulfides in erythrocytes and plasma according to the recently published procedure with minor modifications [21].

#### 2.3.1 Low Molecular Mass Thiols (LMM‐SH) in Red Blood Cells (RBCs)

0.2 mL of blood was washed three times with saline, and after removal of the supernatant, 1 mL of 10 mM phosphate buffer pH 7.4 was added to hemolyze the erythrocytes. An aliquot of the hemolysate was used for the determination of hemoglobin (Hb), while 0.2 mL of the sample was treated with 1 mM monobromobimane (mBrB, final concentration) for 10 min and stored at −80°C until analysis.

#### 2.3.2 Disulfides in RBCs and Plasma

800 μL of blood was transferred to tubes containing 80 μL of 310 mM N‐ethylmaleimide (NEM) and gently shaken for 1 min. The sample was then centrifuged at 8000 *g* for 30 s. The supernatant was collected and stored for analysis of disulfides in plasma. Three hundred microliters of erythrocytes were collected from the bottom of the tube, washed three times with saline, and then hemolyzed by adding 1 mL of 5 mM phosphate buffer, pH 6.5, with 2 mM NEM. The supernatant obtained by centrifugation at 20 000 g for 15 min at 4°C was stored at −80°C for analysis of glutathione disulfide (GSSG) and S‐glutathionylated hemoglobin (HbSSG). For the analysis of membrane‐bound *S*‐thiolated proteins (mRSSP), the pellets were resuspended with a glass rod in 5 mM phosphate buffer, pH 6.5, with 1 mM NEM and centrifuged at 20 000 *g* for 15 min at 4°C; this step was repeated three times. The samples were stored at −80°C until analysis [24].

#### 2.3.3 Plasma Thiols

500 μL of blood was treated with 50 μL of the citrate buffer solution (see Section 2.1). Plasma was collected by centrifugation at 10 000 *g* for 20 s and then 0.2 mL was spiked with 0.2 mL H_2_O and 20 μL of the thiol stabilizing solution and stored at −80°C until analysis.

#### 2.3.4 Protein Inter‐ and Intra‐Chain Disulfides (PSSP) in Plasma and RBCs

500 μL of blood was immediately spiked with 50 μL of 310 mM NEM and shaken for 1 min. Plasma was separated by centrifugation (8000 *g* for 30 s) and purified erythrocytes were obtained by repeated washing of the pellet with saline as described above. 0.1 mL‐aliquots of both samples were added with trichloroacetic acid (TCA, 3% [w/v] final concentration) and stored at −80°C until analysis.

#### 2.3.5 Other Oxidative Stress Parameters

The remaining blood was centrifuged at 8000 *g* for 30 s and the plasma was divided into 0.2 mL aliquots for analysis of protein carbonyl (PCO), malonyldialdehyde (MDA) and F2‐isoprostanes and stored at −80°C until analysis. The aliquot used for F2‐isoprostanes was treated with 0.005% (final concentration) butylated hydroxytoluene before storage.

### 2.4 Blood Treatment for the In Vitro Experiments With Oxidizing Agents

Twenty milliliters of whole blood were collected from the antecubital vein of 5 healthy donors who participated in the clinical study. The blood was collected in evacuated plastic tubes containing K_3_EDTA and treated under controlled conditions (i.e., 37°C temperature, low pO_2_). A stock of glucose (1 M) and glucose oxidase (10 U/mL), both dissolved in saline, was added to the blood to a final concentration of 10 mM and 0.01 U/mL, respectively. This system is known to release hydrogen peroxide [25]. During the experiment, both *tert*‐butyl hydroperoxide (*t*‐BOOH, 10 mM solution in saline) and peroxynitrite (2 mM solution in saline with 10 mM NaOH) were slowly added to the reservoir using a syringe precision pump set to 2 μL/min. The blood flow was set to 20 mL/min. At various time points, 0.5 mL of blood was withdrawn, derivatized for analysis of GSSG, HbSSG, *S*‐thiolated proteins (RSSP), mRSSP in RBCs, the ratio of low molecular mass thiols to low molecular mass disulfides (LMM‐SH/LMM‐SS), protein thiolation index (PTI), PCO and MDA in plasma, and stored at −80°C until analysis as indicated above.

### 2.5 In Vitro Experiments to Evaluate GSH Release From Cells

Five healthy donors who participated in the clinical study (3 women, 2 men) had six milliliters of whole blood collected from the antecubital vein in evacuated plastic tubes containing K3EDTA.

The blood components were obtained by centrifugation under different conditions. In detail: RBCs were purified by centrifuging 0.5 mL of blood at 10 000 *g* for 30 s, removing the supernatant and washing the pellets three times with saline. The RBCs were then diluted with phosphate‐buffered saline pH 7.4 with 5 mM glucose at a hematocrit of 25% and used for the experiments.

Polymorphonuclear leukocytes (PMN) and lymphocytes were separated from 5 mL of blood using PolymorphoprepTM (Fisher Scientific). According to the instructions for use, after centrifugation of the blood, the cells separated into two bands corresponding to PMN and lymphocytes plus monocytes, respectively. The cells were collected and resuspended in PBS and glucose (as above).

Erythrocytes, lymphocytes and polymorphonuclear cells (PMN) were incubated at 37°C for 2 h. The supernatant was then collected by centrifugation (10 000 *g* for 30 s), treated with mBrB and stored at −80°C.

#### 2.5.1 Cell Cultures

Human umbilical vein endothelial cells (HUVEC) were isolated from umbilical cords of uncomplicated pregnancies and grown on 1% (w/v) gelatin in EBM‐2 medium (Lonza). A549 (human lung carcinoma cells), IMR90 (human embryonic lung fibroblasts), HEK293 (human embryonic kidney cells), A375 (human melanoma cells) and HeLa cells were obtained from ATCCs (American Type Culture Collection Manassas, VA). All cells were maintained in DMEM supplemented with 10% heat‐inactivated fetal bovine serum, 100 U penicillin, 100 μg/mL streptomycin, and 2 mM glutamine. All cells were cultured in an atmosphere of 95% air and 5% CO_2_ at 37°C in 100 mm culture plates. After reaching confluence, the culture medium was changed and the cells were harvested after 24 h, deproteinized, treated with mBrB and stored at −80°C.

### 2.6 In Vitro Treatment of Plasma

Six milliliters of whole blood were collected from the antecubital vein of 3 healthy donors (2 females and 1 male) in evacuated plastic tubes containing K_3_EDTA. The samples were immediately centrifuged at 10 000 *g* for 30 s to separate the plasma. The resulting plasma was then pooled, divided into three aliquots, and incubated at 37 °C with different concentrations of GSH (5, 10, and 20 μM). In addition, 1 mM NADPH (final concentration) and 0.5 mL of glutathione reductase were added to the incubation mixture. At 0, 1, 2, 6 and 12 h, 0.1 mL of plasma was collected and stored at −80 °C until analysis of HcySSP.

### 2.7 Analysis of Thiolome, PCO, MDA and F2‐Isoprostane

#### 2.7.1 LMM‐SH in RBCs

Samples in mBrB were deproteinized by treatment with 10 μL 60% (w/v) TCA and centrifugation (2 min at 14 000 *g*). The LMM‐SH were measured in the supernatant by HPLC [21].

#### 2.7.2 Disulfides in RBCs

GSSG was measured in the supernatant after deproteinization of the sample with TCA (0.12 mL hemolyzed sample in NEM + 10 μL 60% [w/v] TCA). Five hundred microliters of the hemolyzed sample in NEM was passed through PD10 columns for gel filtration, and the eluate was used for both Hb determination and HbSSG analysis [26]. GSSG, HbSSG, and mRSSP were measured by HPLC after reduction with dithiothreitol (DTT) and labeling of the released thiol by mBrB [21, 24].

#### 2.7.3 Thiols in Plasma

Protein thiols (PSH) were measured on the spectrophotometer by colorimetric reaction with Ellman reagent (DTNB) [27]. LMM‐SH were measured in the supernatant by fluorometric HPLC after plasma deproteinization with 60% (w/v) TCA and thiol labeling with mBrB [21].

#### 2.7.4 Disulfides in Plasma

0.1 mL plasma was deproteinized by treatment with 10 μL 60% (w/v) TCA. After centrifugation (2 min at 14 000 *g*), the supernatant was used to measure LMM‐SS and the pellet for RSSP analysis. In both cases, the thiols released by DTT treatment were labeled with mBrB and detected by HPLC [21]. The PTI was calculated as the molar ratio between the RSSP and the PSH in plasma [28].

#### 2.7.5 PSSP in Plasma and RBCs

Samples in TCA were centrifuged at 10 000 *g* for 2 min and the supernatant discarded. The pellets were resuspended in 1 mL of 1.5% (w/v) TCA using a glass rod, centrifuged at 10 000 *g* for 2 min and the supernatant discarded. This step was repeated three times. The protein pellet was finally resuspended in 1 mL of 0.2 M Tris–HCl buffer pH 8.0 in the presence of 5 mM DTT. The samples were incubated for 30 min with rotary shaking, then the proteins were precipitated by adding TCA (3% w/v final concentration) and washed several times until DTT disappeared from the supernatant (checked spectrophotometrically with DTNB). The protein pellets were then resuspended in 100 mM Tris buffer pH 8.5 containing 1% SDS with rotary shaking. The PSH released by the reduction of high molecular mass disulfides (HMM‐SS) were measured spectrophotometrically using a spectrophotometer by reaction with DTNB. The PSSP were calculated as the difference between HMM‐SS and RSSP [29].

#### 2.7.6 PCO

Carbonylated proteins were derivatized with 2,4‐dinitrophenylhydrazine (DNPH) and detected by Western immunoblotting with anti‐DNP antibodies according to Colombo et al. [30]. In brief, 400 μg (1 mg/mL) of plasma proteins were mixed with 80 μL of 10 mM DNPH and incubated in the dark for 60 min. Samples were then mixed with 480 μL of 20% (w/v) TCA and incubated on ice for 10 min. After centrifugation at 20 000 *g* for 15 min at 4°C, the protein pellets were washed three times with ethanol: ethyl acetate in a 1:1 ratio to remove free DNPH. The dried protein pellets were resuspended in 2× reducing Laemmli sample buffer. Proteins were separated by SDS‐PAGE on 7.5% (w/v) Tris–HCl polyacrylamide gels, transferred to a PVDF membrane, and detected by Western immunoblotting. Rabbit anti‐DNP (Molecular Probes) was used as the primary antibody. HRP‐conjugated goat anti‐rabbit IgG was used as a secondary antibody (Molecular Probes). The immunoreactive protein bands were visualized by ECL detection.

#### 2.7.7 MDA

MDA was measured in the supernatant obtained by treating 0.4 mL of plasma with 40 μL 60% (w/v) TCA. Two hundred microliters (200 μL) of the supernatant was then reacted with 0.6% (w/v) thiobarbituric acid (TBA) in a 1:1 ratio in a boiling water bath in tightly sealed glass tubes for 10 min. The samples were then cooled in an ice‐water bath and immediately analyzed by colorimetric HPLC [31].

#### 2.7.8 F_2_ ‐Isoprostanes (8‐iso‐PGF2a)

Free 8‐isoprostanes were measured in plasma using a commercial ELISA kit (Cayman) that utilizes an 8‐isoprostane‐acetylcholinesterase conjugate as a competitor for the binding sites in 8‐isoprostane‐specific rabbit antibody binding sites. Plasma samples were purified by solid‐phase extraction and then read in a 96‐well microplate reader at a wavelength of 420 nm according to the manufacturer's instructions.

### 2.8 Protein Determination

The cell protein concentration was measured in acid‐precipitated pellets according to the Bradford assay [32] after they had been dissolved in 0.2 mL 0.1 N NaOH.

Hb concentration was measured in an aliquot of hemolyzed RBCs using a spectrophotometer (wavelength range 500–700 nm) [33].

### 2.9 Statistics

The data are given as mean ± SD. The differences between the mean values were determined using ANOVA followed by a Bonferroni post‐test. A value of *p* < 0.05 was considered statistically significant. Normality was tested using the Kolmogorov–Smirnov test. Correlation analysis was performed by calculating Pearson's product–moment correlation coefficient. Holm‐Bonferroni was used to adjust for multiple comparisons.

## 3 Results

### 3.1 Clinical Study

This study focuses on the question of whether fluctuations in plasma homocysteine levels in healthy individuals can be associated with the distribution of high and low molecular weight thiols and disulfides in intra‐ and extracellular compartments. To this end, the different redox forms of high and low molecular mass thiols were measured in the blood of healthy subjects (what we can call the blood thiolome). As numerous factors can influence the plasma levels of all aminothiols present, we decided to include a carefully selected group of men and women in the study (see Section 2.2 for details) who were particularly homogeneous in terms of age, lifestyle, and dietary habits, had no chronic diseases, and had not taken any medication or supplements in the previous 6 months. The information on the group of people included in this study is shown in Table 1. The data on the thiolome are summarized in Table 2.

**TABLE 2:** Thiolome in blood of healthy human volunteers. Data are reported as mean ± SD, *n* = 62.

Parameter	Intraerythrocitic μM	Extracellular μM
GSH	2463 ± 174	2.64 ± 0.52
GSSG	3.69 ± 0.03	1.11 ± 0.33
GSH/GSSG	821 ± 168	1.88 ± 0.52
GSSP	2.74 ± 0.34	0.73 ± 0.09
Cys	18.4 ± 0.99	12.3 ± 0.56
CySS	Nd	59.8 ± 8.40
Cys/CySS	—	0.11 ± 0.06
CySSP	Nd	113 ± 39
CysGly	Nd	2.44 ± 0.09
CySSGly	Nd	5.60 ± 0.21
CysGly/CySSGly	—	0.41 ± 0.01
CyGlySSP	Nd	5.04 ± 0.66
Hcys	8.73 ± 0.33	0.183 ± 0.061
HcySS	Nd	1.12 ± 0.045
Hcys/HcySS	—	0.042 ± 0.007
HcySSP	Nd	10.3 ± 2.66
γ−GluCys	13.8 ± 0.9	0.052 ± 0.003
γ−GluCySS	Nd	0.821 ± 0.102
γ−GluCys/γ−GluCySS	—	—
γ−GluCySSP	Nd	1.32 ± 0.12
PSH	9840 ± 1566	424 ± 83
PSSP^a^	25.3 ± 1.1	14 850 ± 2430
mRSSP	4.05 ± 0.22	—
RSSP	5.95 ± 0.85	125 ± 29
PTI		0.395 ± 0.258
LMM‐SH/LMM‐SS	821 ± 166	0.217 ± 0.081
tSH/tSS	123 340. ± 121	0.0294 ± 0.005

Abbreviations: CyGlySSP, mixed disulfide between cysteinylglycine and proteins; Cys, cysteine; CysGly, cysteilylglycine; CySS, cystine; CySSGly, cysteinylglycine disulfide; CySSP, *S*‐cysteinylated proteins; GSH, reduced glutathione; GSSG, glutathione disulfide; GSSP, S‐glutathionylated proteins; Hcys, reduced homocysteine; HcySS, homocystine; HcySSP, S‐homocysteinylated proteins; LMM‐SS/LMM‐SS, low molecular mass thiols/low molecular mass disulfides; mRSSP, membrane RSSP; PSH, protein thiols; PSSP, protein inter and intra‐chain disulfides; PTI, protein thiolation index; tSH/tSS, total thiols/total disulfides; γ−GluCys, γ−glutamylcysteine; γ−GluCySS, γ−glutamylcystine; γ−GluCySSP, mixed disulfide between γ−glutamylcysteine and proteins.

^a^ Intraerytrocytic RSSP = cytosolic + membrane RSSP; extracellular RSSP = GSSP + CySSP + CyGlySSP + HcySSP + γ−GluCySSP.

It is known that thiols and disulfides are distributed differently between intracellular and extracellular compartments [34]. Specifically, in blood we found a ratio between PSH and PSSP of > 100 in the cytoplasm and < 0.003 in plasma, as well a ratio between LMM‐SH and LMM‐SS > 800 intracellularly and < 0.25 in plasma. Large amounts of mixed disulfides between proteins and LMM‐SH were present in plasma but almost absent in cells. A variable distribution of the different LMM‐SH (and LMM‐SS) between the two compartments was also evident, with the intracellular environment being extremely rich in reduced glutathione (GSH), while extracellularly cysteine (Cys) predominates over the other LMM‐SH, but only by an order of magnitude (or less). The LMM‐SS and RSSP redox forms for each aminothiol (except GSH) predominate over the reduced forms. In particular, the ratios between free thiols, disulfides, and mixed disulfides with proteins are relatively similar for cysteine (1:4:7) and cysteinylglycine (1:2:2), whereas for glutathione and homocysteine they are clearly shifted towards the reduced and oxidized forms, respectively (glutathione, 1:0.4:0.2; homocysteine, 1:12:56). It is striking that homocysteine is the only aminothiol that occurs mainly as a mixed disulfide bound to proteins (HcySSP, about 80%–85%), while all other aminothiols are bound to less than 50%.

**FIGURE 1:**
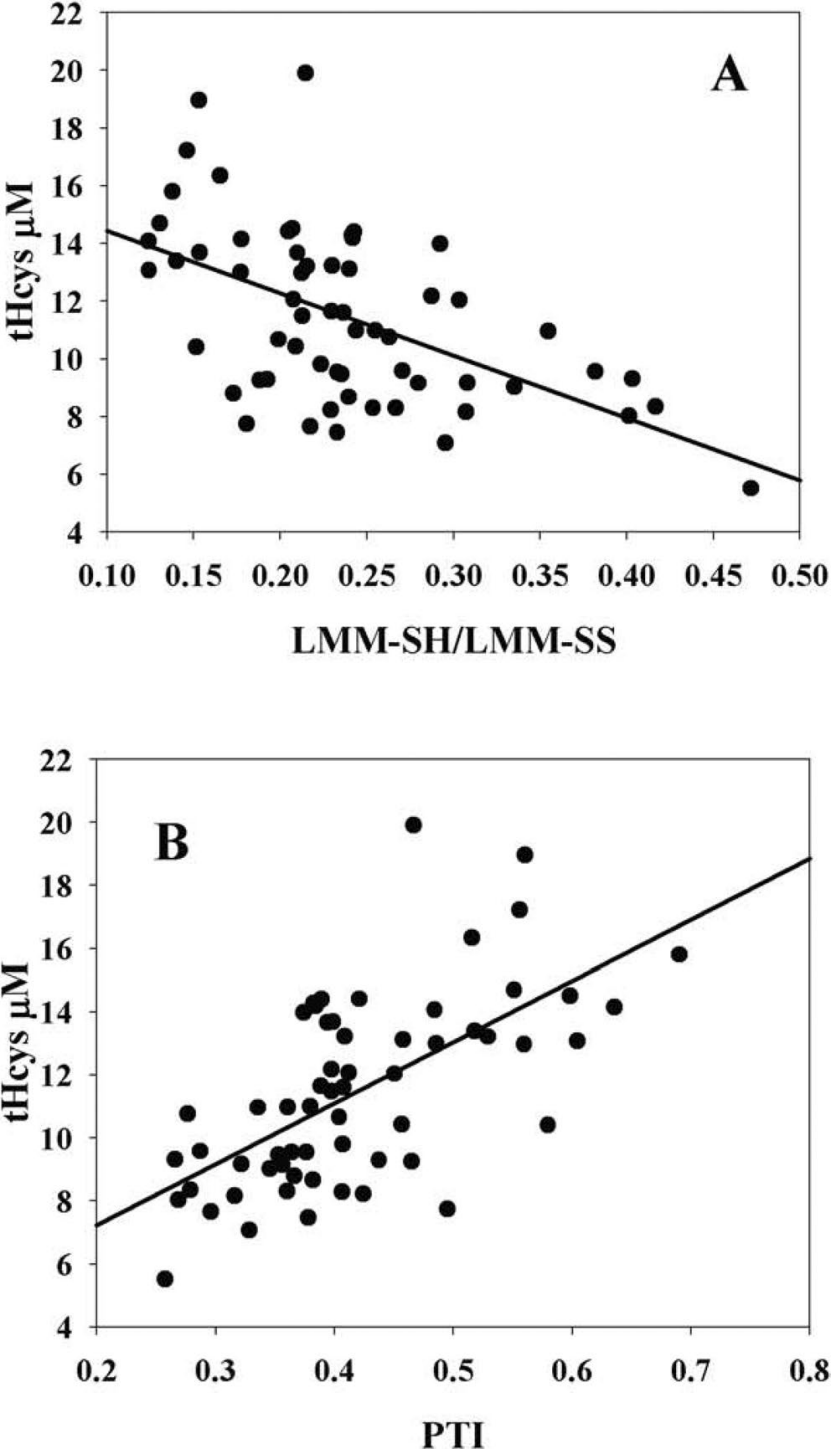
Correlations between tHcys and thiol to disulfide ratios in plasma. Total homocysteine was calculated as the sum of Hcys + 2xHcySS + HcySSP using the data summarized in Table 2. The values refer to the clinical study carried out on 62 healthy volunteers.

Although homocysteine is present in plasma in various redox forms, for diagnostic/prognostic purposes its sum is routinely measured, which should be referred to as total Hcys (tHcys). In addition to the reduced form (Hcys), this parameter also includes the disulfide forms (HcySS and HcySSP). In our study, tHcys values were within the normal range (< 15 μM) in most of the subjects studied (~88%), with only a small percentage of subjects exhibiting mild hyperhomocysteinemia; this is consistent with values reported for a 30–50 year old population [6]. The tHcys values showed an inverse correlation with the LMM‐SH/LMM‐SS ratio (*r* = −0.5152, *p* < 0.05) and a direct correlation (*r* = 0.6284, *p* < 0.01) with the PTI (which is calculated as the ratio RSSP/PSH in plasma) (Figure 1A,B). This means that the higher the thiol content in the reduced form, the lower tHcys is.

To test whether different variables, in particular the individual oxidative state, could play a role in the observed correlations, we analyzed a handful of different biomarkers for oxidative stress in all subjects of the present study and correlated them with tHcys. Therefore, in addition to the thiol oxidation parameters listed in Table 2, we also analyzed several biomarkers of lipid (MDA, F_2_‐isoprostanes) and protein oxidation (PCO). No correlation was found between these biomarkers of oxidative stress and tHcys. There was also no correlation between these biomarkers and thiols, disulfides and the ratio of thiols to disulfides in the plasma environment (Table 3). No correlation was also found between tHcys and the GSH/GSSG ratio in RBCs or the concentration of *S*‐thiolated proteins in erythrocyte membranes (mRSSP). In contrast, both GSH/GSSG and mRSSP, which are extremely sensitive biomarkers of oxidative stress [24, 26], correlate positively with MDA, PCO and F_2_‐isoprostanes.

**TABLE 3:** Pearson‘s correlation matrix of blood thiols, disulfides and other biomarkers of oxidative stress measured in clinical study. Parameters were measured in red blood cells (RBC) and in plasma.

Parameter	RBC GSH	RBC GSSG	RBC GSH/GSSG	HbSSG	RBC mRSSP	Cys	CysGly	GSH	GSH + CysGly	LMM‐SH/LMM‐SS	HcySSP	Hcys/HcySSP	tHcys	PTI	MDA	F2‐isoprostans
RBC GSSG	−0.18															
RBC GSH/GSSG	0.123	0.752														
HbSSG	−0.124	−0.068	0.091													
RBC mRSSP	0.084	0.479	0.774	−0.124												
Cys	0.032	0.122	−0.132	0.154	0.011											
CysGly	−0.142	0.268	−0.225	−0.011	−0.120	0.402										
GSH	0.255	−0.041	0.266	−0.229	0.038	0.445	0.404									
GSH + CysGly	0.255	0.106	0.145	0.033	0.052	0.545	0.763	0.846								
LMM‐SH/LMM‐SS	0.058	−0.017	0.019	−0.051	0.068	0.697	0.592	0.664	0.776							
HcySSP	0.055	−0.133	0.053	0.037	−0.044	−0.280	−0.446	−0.453	−0.514	−0.332						
fHcys/HcySSP	0.076	−0.150	0.182	0.083	−0.052	0.105	−0.050	0.264	−0.265	−0.188	−0.569					
tHcys	0.017	−0.077	−0.010	−0.007	−0.070	−0.351	−0.608	−0.589	− 0.689	0.515	0.906	−0.399				
PTI	−0.214	0.045	0.074	0.015	0.011	0.453	−0.586	−0.431	−0.596	−0.748	0.425	−0.287	0.628			
MDA	0.056	0.362	−0.344	0.239	−0.285	0.125	0.129	−0.074	0.230	−0.041	−0.133	0.103	−0.115	0.235		
F2‐isoprostans	0.050	0.063	0.301	−0.110	0.422	0.043	0.118	−0.113	0.166	−0.057	0.158	0.098	0.109	0.171	0.226	
PCO	0.132	−0.012	0.286	−0.045	0.318	−0.054	0.033	−0.087	−0.094	−0.023	0.039	−0.164	0.084	0.118	0.194	0.087

*Note:* *p* < 0.05; *p* < 0.01; *p* < 0.001.

Abbreviations: Cys, cysteine; CysGly, cysteilylglycine; fHcys/HcySSP, free Hcys/S‐homocysteinylated proteins; GSH, reduced glutathione; GSSG, glutathione disulfide; HbSSG, S‐glutathionylated hemoglobin; HcySSP, S‐homocysteinylated proteins; LMM‐SS/LMM‐SS, low molecular mass thiols/low molecular mass disulfides; MDA, malonyldihaldehyde; mRSSP, membrane S‐thiolated proteins; PCO, protein carbonyls; PTI, protein thiolation index; tHcys, total homocysteine.

**FIGURE 2:**
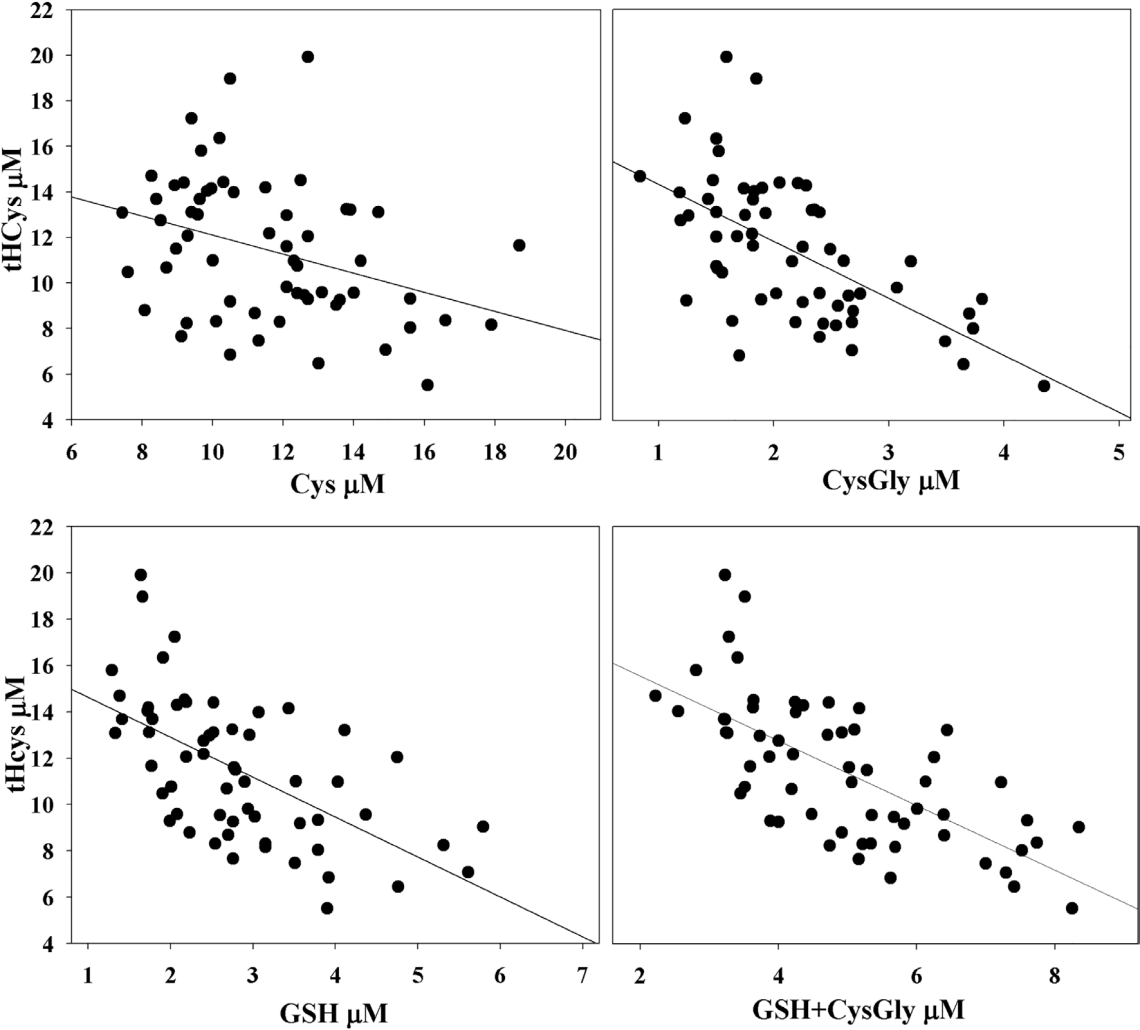
Correlations between tHcys and LMM‐SH in plasma. Total homocysteine was calculated as the sum of Hcys + 2xHcySS + HcySSP using the data summarized in Table 2. The values refer to the clinical study carried out on 62 healthy volunteers.

Based on the observed inverse correlation between tHcys levels and LMM‐SH (Figure 1) we then investigated whether specific correlations exist with different plasma thiols or disulfides (Table 3 and Figure 2). An inverse correlation of tHcys with GSH (*r* = −0.589, *p* < 0.001), cysteinilglycine (CysGly, *r* = −0.608, p < 0.001), GSH + CysGly (*r* = −0.686, *p* < 0.001) was found. In addition, it is noteworthy that there was a highly significant inverse correlation was present between GSH + CysGly and HCySSP (*p* < 0.001).

### 3.2 In Vitro Experimental Model of Oxidative Stress in Blood

Based on the data presented in Table 3, two main observations can be made: (i) there is no apparent correlation between total homocysteine (tHcy) levels and oxidative stress markers; and (ii) there is an inverse correlation between glutathione and tHcy. These results prompted us to investigate in an in vitro model whether mild oxidative stress could be associated with increased tHcy levels. As homocysteine is predominantly present in the form of protein‐bound homocystine (hCySSP), an increased presence of reactive oxygen species and thus oxidative stress could promote the formation of disulfide bonds between homocysteine and plasma proteins.

**FIGURE 3:**
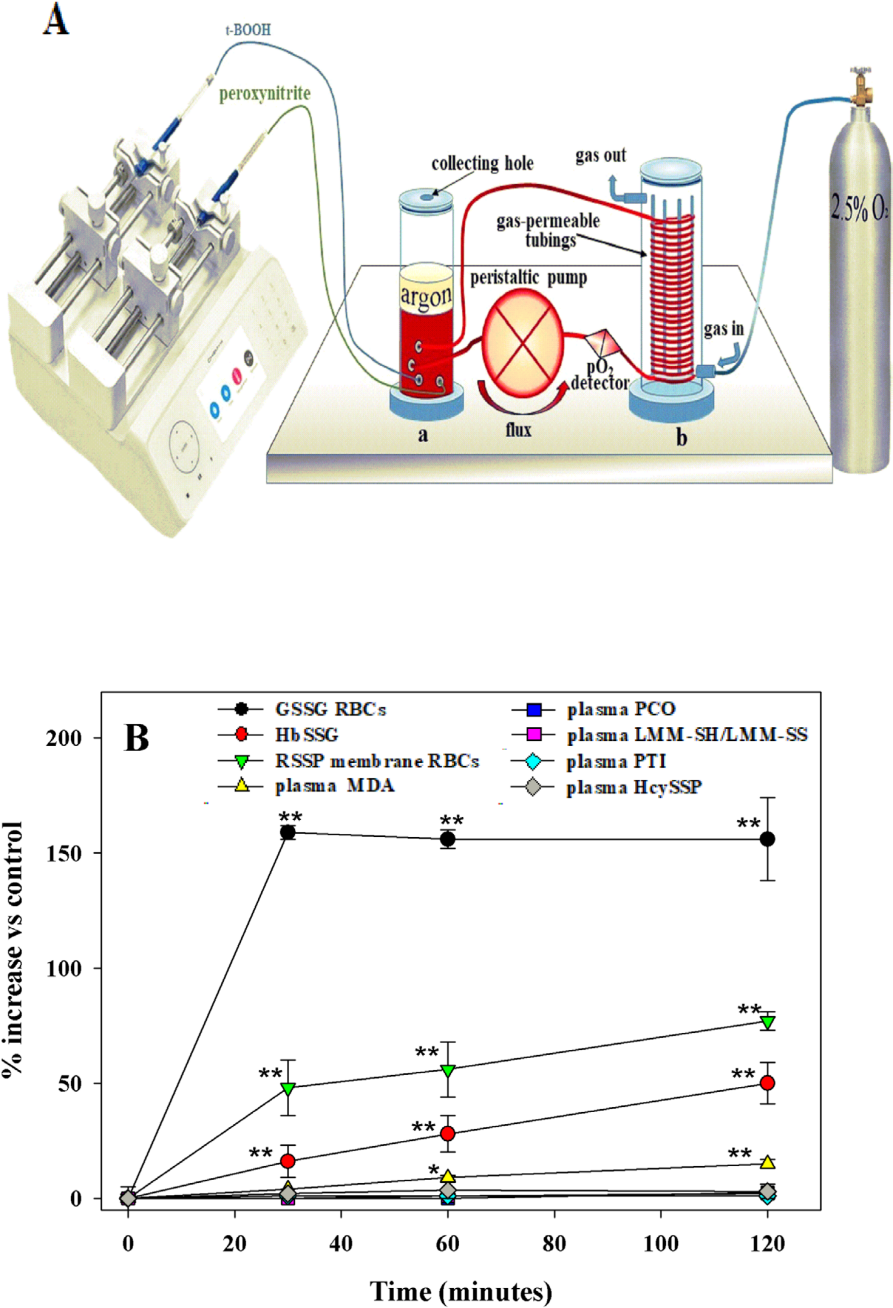
Evaluation of different biomarkers of oxidative stress in human blood. Human blood was continuously exposed to oxidants (glucose/glucose oxidase, t‐BOOH, peroxynitrite) under controlled conditions (37°C, blood flow 20 mL/min, 2.5% O_2_/97.5% N_2_) using a device shown schematically in (A). The device consists of two cylinders: (a) blood chamber, (b) gas equilibration chamber circulated with 2.5% O_2_, 97.5% N_2_. Blood is fluxed (20 mL/min) throughout the two cylinders by a peristaltic pump. A double syringe precision pump delivers the oxidants into the blood‐containing cylinder. The temperature is controlled by placing the device in a thermostatically controlled cabinet. (B) At time 0 (before oxidant treatment) and after 30, 60 and 120 min of continuous exposure to oxidants aliquots (0.5 mL) of blood were collected for analysis of the indicated biomarkers of oxidative stress. *n* = 5. **p < 0.01 versus time 0; * < *p* < 0.05 versus time 0.

Human blood was treated with a slow but continuous delivery of minimal amounts of oxidizing agents (glucose/glucose oxidase, t‐BOOH and peroxynitrite) using a special device (Figure 2A) that allows the rate of delivery of the oxidizing agents to be adjusted while generating a continuous blood flow. We have reproduced here a slow release of oxidants, as should generally occur in vivo from the surrounding tissues to the blood. Blood pO_2_ tension and temperature were also tightly controlled. A pro‐oxidizing environment was generated introducing a flow of 2.5% pO_2_ which produced a 40% oxygenated Hb. Under these conditions, hemoglobin becomes slightly leaky and generates the anion superoxide [35]. In this experiment, we observed a marked rise in some oxidative stress biomarkers, especially intracellular ones (GSSG, mRSSP and HbSSG), which increased significantly at the first time point of analysis (30 min) and remained high throughout the experiment (120 min). MDA also increased, albeit slowly and to a lesser extent. In contrast, all other parameters measured in the plasma, especially thiols and disulfides, remained unchanged (Figure 3B). Moreover, the effect of the oxidizing agents on HcySSP levels was negligible. Overall, these results suggest that even under conditions of systemic oxidative stress, plasma biomarkers, including HcySSP, remain largely unaffected. This supports the hypothesis that plasma is not the primary target of oxidative stress; instead, reactive oxygen species likely act primarily on intracellular thiol‐containing compounds.

### 3.3 Export of GSH From Cells

**FIGURE 4:**
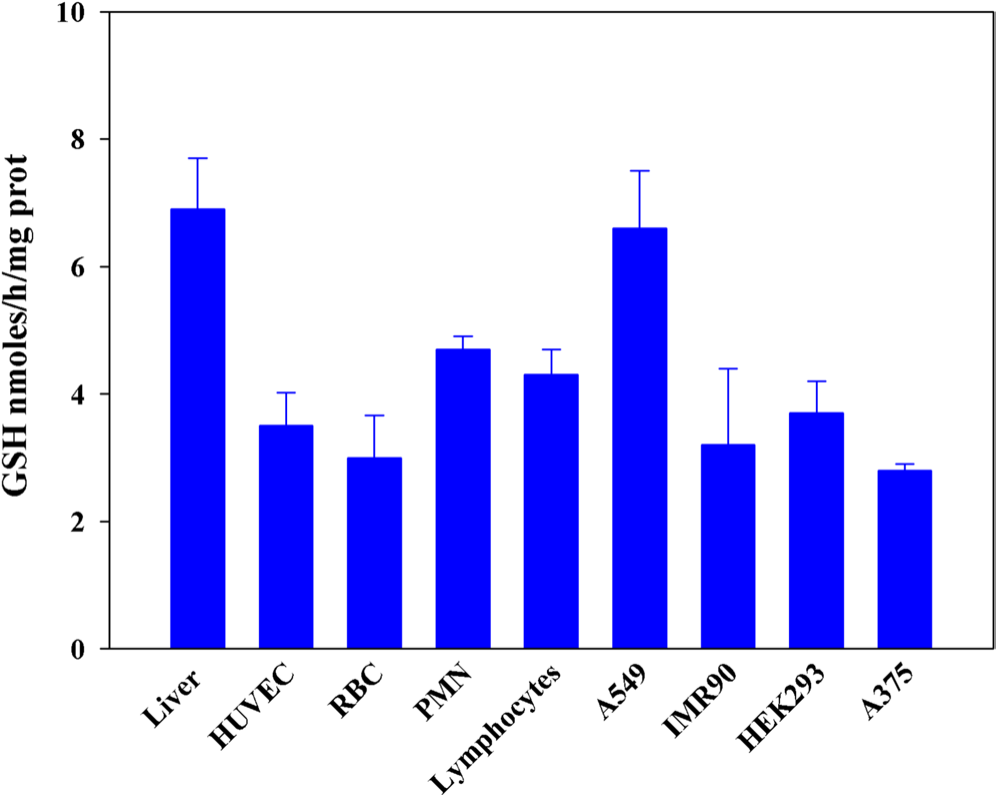
Release of GSH from various cell types and tissues. GSH was measured in the extracellular medium after a 24‐h incubation of cultured cells at confluence. RBCs, lymphocytes, and polymorphonuclear cells (PMN) were purified from human blood and, after a 2‐h incubation at 37°C, GSH was measured in the extracellular milieu. *n* = 5 for each cellular model. For comparison, our data on release from the liver, obtained with the isolated perfused organ model and previously published, were included [36].

The contribution of the liver, blood cells, and various types of cultured cells to GSH export was then evaluated. The cells were cultured for 2 h, and the data were compared with those previously obtained in our laboratory using the isolated perfused rat liver. As shown in Figure 4, GSH is consistently exported from all cells examined with the liver exporting the most. GSH release from red blood cells amounted to about 15% of their content.

### 3.4 In Vitro Treatment of Plasma With GSH

**FIGURE 5:**
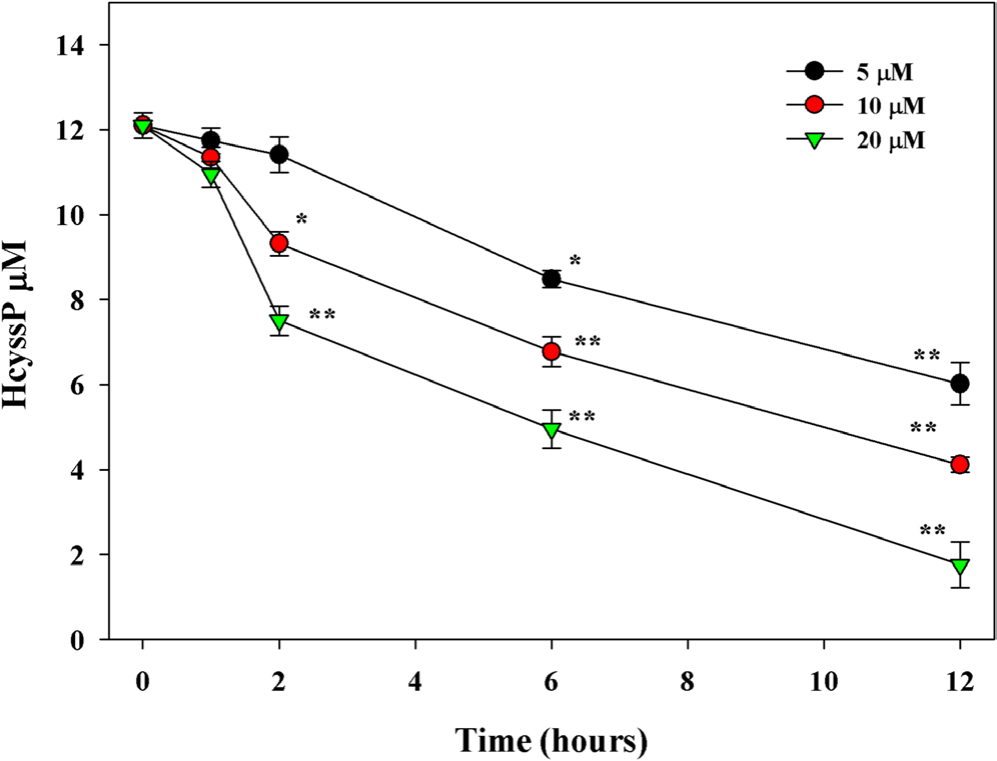
Time course of HCySSP from 3 pooled plasma samples (plasma initial levels of HCySSP: 15.6, 12.2, 8.6 μM) after in vitro treatment with increasing concentrations of GSH (5, 10, 20 μM) in the presence of 2 mM NADPH and 0.5 U/mL glutathione reductase. *n* = 3. **p < 0.01 versus time 0; * < *p* < 0.05 versus time 0.

To investigate the effect of thiol‐disulfide exchange on Hcyssp levels, we performed an in vitro experiment with plasma samples from three healthy volunteers. The samples were treated with GSH at three different concentrations, and to promote the reductive environment required for dethiolation, we also added NADPH and glutathione reductase. This arrangement should favor the reduction of disulfide bonds and shift the equilibrium towards the formation of free thiol groups. The time‐dependent change in HcySSP concentrations is shown in Figure 5. The data indicate that the dethiolation of HcySSP proceeds at a relatively slow but concentration‐dependent rate, as expected for this type of redox reaction. Furthermore, the extent and kinetics of the process appear to be directly influenced by the initial GSH concentration added to the system, highlighting its key role in modulating the thiol‐disulfide equilibrium under these conditions.

### 3.5 Correlation Between tHcys and GSH + CysGly Levels in Plasma of Different Mammals

**FIGURE 6:**
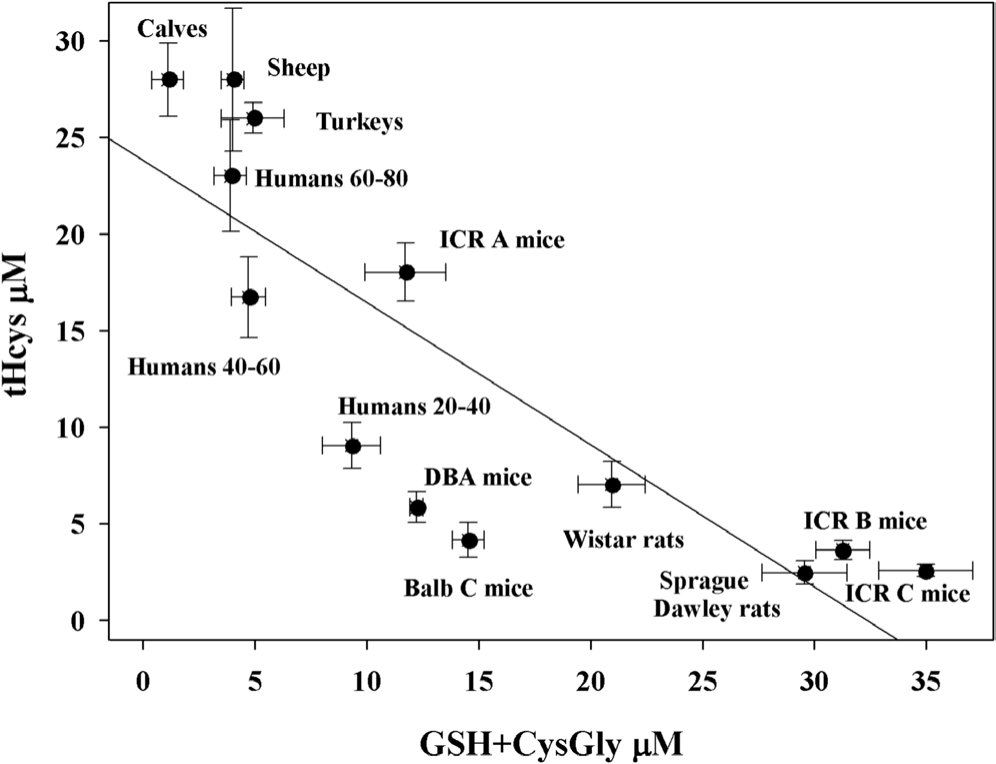
Inverse correlation between GSH + CysGly and tHCys content in plasma of different mammals. The data for laboratory animals were obtained in our laboratory and published previously [38, 39, 40, 41]. ICR mice are classified into three subgroups (A, B, C) on the basis of phenotypic and genetic differences [40]. Values for humans include original data from this work and previous data [37]. Calves: *N* = 11; sheep: *N* = 9; turkeys: *N* = 15; ICR A mice: *N* = 18; ICR B mice: *N* = 16; ICR C mice: *N* = 16; DBA mice: *N* = 26; BALB C mice: *N* = 26; Wistar rats: *N* = 18; Sprague–Dawley rats: *N* = 32; humans 20–40: *N* = 67; humans 40–60: *N* = 75; humans 60–80: *N* = 32.

Plasma levels of total homocysteine (tHcy) were analyzed in different animal species to investigate their possible correlation with GSH + CysGly concentrations. Human subjects were divided into three age groups (20–40, 40–60, and 60–80 years). Two of these groups (20–40 and 40–60 years) included also participants who took part in the present clinical study. The other data came from our previous research with healthy individuals of different ages [28, 37]. Plasma data previously collected in our laboratory in different mammalian species such as mice, rats, calves, sheep, and turkeys were also used for comparison [38, 39, 40, 41]. The mean tHcys concentrations varied significantly in the groups studied and showed a strong inverse correlation with GSH + CysGly levels (*r* = −0.829, *p* = 0.0005; Figure 6). Plasma concentrations of GSH + CysGly vary considerably among the animal species studied, with some rodents (e.g., ICR B and ICR C mice) showing particularly high levels (> 30 μM). In contrast, other mammals such as calves and sheep have remarkably low levels of GSH and CysGly. This corresponds to a broad spectrum of tHcys concentrations in plasma, ranging from nearly 30 μM in calves and sheep (where GSH and CysGly are present in minimal amounts) to 1–2 μM in ICR mice (where the concentrations of GSH and CysGly is much higher). In humans, the age‐related decline of GSH and CysGly in plasma is accompanied by an increase in tHcys [6, 37, 42], a trend that was also confirmed by our results.

## 4 Discussion

The main finding of this study is that total homocysteine (tHcys) levels in the plasma healthy individuals are closely associated with fluctuations in other low‐molecular‐mass thiols (LMM‐SH), particularly glutathione (GSH). This conclusion is based on a series of analyses performed on blood samples from 62 healthy volunteers. It is noteworthy that homocysteine is the only aminothiol that is predominantly present as a mixed disulfide with proteins (80%–85% of its total plasma concentration, see Table 2). The reason for this peculiarity is not fully understood. In general, the ratio between the different redox forms of plasma aminothiols is determined by the thiol‐disulfide exchange reactions (1), whose kinetics and equilibrium constants are influenced by several factors, but certainly follow the law of mass action. Protein SH can also be involved in these reactions (2):

(1) $$R_{1}\mathrm{SH}+R_{2}\mathrm{SS}R_{3}↔R_{1}\mathrm{SS}R_{3}+R_{2}\mathrm{SH}$$

(2) $$\mathrm{PSH}+R_{1}\mathrm{SS}R_{2}↔\mathrm{PSS}R_{1}+R_{2}\mathrm{SH}$$

Previous studies have attributed an important role to albumin, which is known to possess a Cys residue at position 34 that is partially bound to LMM‐thiols via a disulfide bridge [43]. As the –SH group of this cysteine has a low pKa, it can participate in several reactions at physiological pH, which include the thiol‐disulfide exchange reactions with LMM‐SS, forming mixed disulfides, especially with cysteine (CySSP). It has been hypothesized that reduced homocysteine (Hcys) enters the bloodstream and attacks cysteinylated albumin, making it dethiolated via an uncatalyzed thiol‐disulfide exchange reaction.

The free ‐SH just‐formed in albumin in turn reacts with homocystine (HcySS) or with the mixed disulfide between cysteine and homocysteine to form albumin‐bound homocysteine (HcySSP) [44]. These reactions are favored over other trans‐thiolations both by steric hindrance and by the pKa value of the leaving group. An important point is that the equilibrium constant for the exchange reactions between thiol‐disulfide pairs in plasma does not match those calculated in vitro at neutral pH [43]. The estimation of this parameter for the reactions between different LMM‐SS and the SH group of albumin is difficult to calculate, but again it is assumed that the different thiol‐disulfide pairs are far from equilibrium [45]. In addition, low molecular weight thiols are slowly oxidized to low molecular weight disulfides, mainly by catalysis of ceruloplasmin [46]. In the case of homocysteine, the fact that it is predominantly covalently bound to plasma proteins as a mixed disulfide is an important factor in its pharmacokinetics, as it can hardly be eliminated.

We have considered the possibility that oxidative stress may play a role in determining the HcySSP content. Indeed, the extracellular compartment is essentially devoid of antioxidants [45], and even a minimal increase in oxidative stress could decrease circulating thiols and generate more disulfides. The shifts in balances leads to the formation of the poorly eliminable albumin‐bound homocysteine, so oxidative stress could be the cause of possible hyperhomocysteinemia. However, the oxidative stress parameters measured in the clinical study showed no correlation with the redox forms of Hcys (Table 3). These results suggest that: (i) oxidative stress has not effect on plasma total homocysteine (tHcys); and (ii) its effects are probably mainly confined primarily to the intracellular environment. These observations were confirmed by data from an in vitro experiment in which human blood was treated with oxidants and intracellular and extracellular biomarkers of oxidative stress were analyzed (Figure 3). The results indicate that intracellular antioxidants face mainly with reactive oxygen/nitrogen species (RO(N)S) and that the extracellular milieu plays only a minimal, if any, antioxidant role. This also supports the concept that plasma levels of tHcys and the ratios between the different redox forms are not related to the oxidative status in the bloodstream.

The steady‐state concentrations and ratios of reduced and oxidized thiols in plasma are influenced by several processes in addition to the kinetics of the thiol‐disulfide exchange reactions (1) and (2). For example, autoxidation of free thiols and the rate of enzymatic degradation and transport s of LMM‐SH/SS across cell membranes are variability factors that contribute to the observed differences in the ratios between the different redox forms of aminothiols in plasma.

Cysteine and glutathione are the most abundant thiols in mammalian tissues, with GSH predominating intracellularly and cysteine in extracellular fluids (see Table 2 and [34]). These two compounds are metabolically linked, and while GSH represents a defense mechanism against RO(N)S‐mediated damage within cells, it certainly has a different role in the extracellular fluids. This hypothesis is underscored by the experiment in Figure 3, in which a huge increase in its oxidized forms following oxidant exposure was found only in the intracellular compartment. GSH is synthesized intracellularly from its amino acids cysteine, glutamate and glycine in a two‐ step reaction catalyzed by the enzymes ‐glutamylcysteine synthetase and GSH synthetase [46]. Cellular cysteine can have various uses: it can be incorporated into GSH and proteins (and other essential molecules such as coenzyme A), it can be metabolized to produce taurine and sulfate, and finally, it can be exported [47]. Extracellular cysteine rapidly oxidizes to cystine (CySS); CySS may have a significant physiological role as a source of Cys because once it enters another cell, it can be reduced back again to Cys by GSH via catalyzed or uncatalyzed thiol‐disulfide exchange reactions [48]. Cystine is transported into cells by several transporters. One of the best studied is the Na^+^‐independent specific cotransporter system Xc^−^ which utilizes the antiporter system for neutral amino acids to exit cells [49]. Extracellular cysteine probably also originates from GSH hydrolysis by the enzyme ‐glutamyltranspeptidase (‐GT), which is located on the extracellular side of the plasma membrane. Specifically, γ−GT converts GSH into CysGly, which in turn is hydrolyzed to Cys and Gly by dipeptidases [50]. The amino acids released by this mechanism (‐GT is widely distributed in animal tissues) can remain in the bloodstream or be taken up by other cells, contributing to thiol homeostasis between organs. Therefore, the uptake of cysteine or cysteine and the intracellular reduction process may play a role in maintaining the plasma thiol‐disulfide balance. GSH is also exported by cells. GSH conjugates are known to be transported outside the cells by multi‐drug resistance proteins (Mrp/ABCC proteins). A total of nine functional Mrp genes have been identified (Mrp1 to Mrp9), and almost all of them transport GSH conjugates [51]. Less information is available on the export of free GSH, but increasing data suggest that most Mrp transporters may also be involved in this process [52, 53]. The export of GSH is common to many, perhaps all, cells, although the liver is considered the major supplier of GSH and its derivatives in plasma [54].

To explore this topic further, we performed the experiment shown in Figure 4, in which the contribution of liver, blood cells and a number of different types of cultured cells to the export of GSH was investigated.

All samples tested showed the ability to release significant amounts of GSH into the extracellular compartment every hour, with the liver making the largest contribution. Small amounts of LMM‐SH other than GSH, particularly Cys and Hcys, are also exported by RBCs [55]. Endothelial cells have also been characterized in the past for their ability to release Hcys [56, 57].

Although the liver is considered the main supplier of plasma GSH for inter‐organ exchange, our data suggest that other cells also play a non‐negligible role and that even poorly metabolizing cells such as RBCs can make a significant contribution. Indeed, in humans, we found a strong direct correlation between plasma thiols and red blood cells count [55].

The strength of the correlations shown in Figure 2 and Table 3 appears to assign a central role to GSH in regulating the SH/SS ratio and thus the level of tHcys in the extracellular fluids. The results of treatment of human plasma with glutathione (GSH) support this observation as they showed a slow but significant decrease in HcySSP levels. This effect was more pronounced at higher GSH concentrations, indicating a clear dose‐dependent relationship (Figure 5).

It should be noted that the sum GSH + CysGly can also be taken into account, as CysGly always results from the enzymatic degradation of GSH. This aspect is confirmed by data from the work of Garibotto et al. [58], where a release of CysGly is reported for peripheral tissues and splanchnic organs, which presumably have GSH‐degrading enzymes in the cell membranes. We therefore investigated whether tHcys correlates with plasma concentrations of GSH + CysGly in different mammalian species. This analysis was motivated by our previous results showing that GSH (and CysGly) concentrations vary considerably between species. It is also known that plasma GSH concentration decreases with age, an observation that our study confirmed in human participants divided into three age groups (Figure 6). Notably, we observed a linear inverse correlation between plasma tHcys and GSH + CysGly concentrations, supporting the hypothesis that homocysteinemia is influenced by fluctuations in plasma GSH.

**FIGURE 7:**
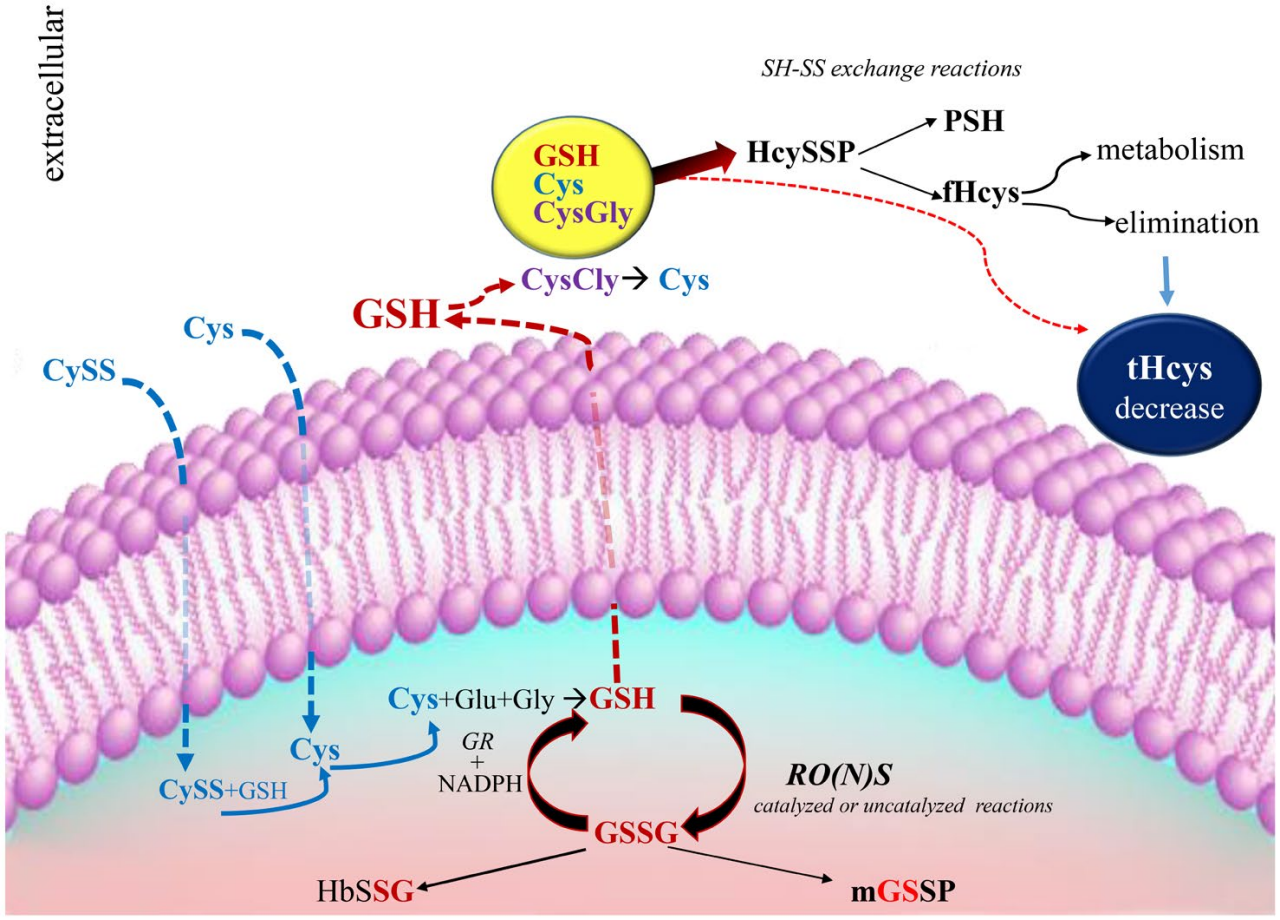
Schematic representation of GSH as defense against RO(N)S (intracellular) and tHcys modulator (extracellular). The rate of GSH synthesis within cells is limited by Cys availability. Both Cys and CySS can enter the cells and are involved in GSH synthesis. GSH can be reversibly oxidized by RO(N)S to GSSG which in turn can cause an increase in both cytoplasmic and membrane *S*‐glutathionylated proteins. The GSH release by the cells is enzymatically degraded producing other LMM‐SH (CysGly and Cys). These LMM‐SH can react with HcySSP (the prevailing form of homocysteine in plasma) generating fHcys. This mechanism makes a large part of the homocysteine available for clearance. Cys, cysteine; CysGly, cysteinylglycine; CySS, cysteine; fHcys, reduced homocysteine + homocysteine disulfide; Glu, glutamate; Gly, glycine; GR, glutathione reductase; GSH, reduced glutathione; GSSG, glutathione disulfide; HbSSG, S‐glutathionylated hemoglobin; HcySSP, S‐homocysteinylated proteins; mGSSP, S‐glutathionylated membrane proteins; RO(N)S, reactive oxygen‐nitrogen species; tHcys, FHcys+ HcySSP.

Dozens of clinical studies have shown that a high concentration of Hcys in plasma is a significant risk factor for mortality, regardless of cause [59]. Retrospective and prospective studies as well as meta‐analyses confirmed that Hcys in plasma is a risk factor for cardiovascular disease (CVD) and stroke [7, 8, 60]. However, the potential role of homocysteine as an enhancer of cardiovascular risk factors has yet to be proven or disproven with certainty, and doubts remain about the causal involvement of hyperhomocysteinemia in atherosclerosis still persist. In particular, the results of large randomized double‐blind trials on the efficacy of homocysteine‐lowering therapy suggest that lowering tHcys levels does not have a beneficial effect on CVD risk, and instead suggest that homocysteine may not play a direct pathogenic role [17, 61, 62]. To complicate matters, epidemiologic data from some countries with prophylactic folic acid fortification of cereal products and results from large primary prevention trials show a reduction in primary stroke events and a decrease in neural tube defects. It is important to remember that vitamin‐based interventions, particularly those containing high doses of folic acid and vitamins B6 and B12, may not be biologically inert. These treatments may potentially have unanticipated adverse effects or interact negatively with existing pathophysiologic conditions, which could negate the expected benefits [20]. In addition, homocysteine may play a more important role in the early stages of cardiovascular disease, when vascular damage is still limited and potentially reversible. In contrast, therapeutic strategies aimed solely at lowering Hcy levels may not effectively alter clinical outcomes in the later stages when atherosclerotic lesions and endothelial dysfunction are already established. The failure of Hcy‐lowering therapies to reduce cardiovascular events and mortality in clinical trials may therefore be due in part to the timing of the intervention and the underestimated complexity of the metabolic pathways involved [20]. To date, the mechanism mediating the harmful effects of homocysteine is only partially understood. In this manuscript, we hypothesize that homocysteine levels should not be considered an independent variable, but are closely related to plasma levels of some specific LMM‐SH. In support of this hypothesis, our results and others emphasize that treatment with NAC, penicillamine, and anethole dithiolethione, which increase plasma GSH levels, resulted in a significant decrease in homocysteine [36, 63, 64]. Furthermore, administration of MESNA (a sulfhydryl compound) to cancer patients, although it does not directly increase plasma GSH, has a strong effect on the plasma thiol/disulfide balance and coincidentally leads to a dramatic decrease in tHcys from 12.3 ± 2.1 to 1.4 ± 1.1 μM [65]. A strong dose‐dependent inverse correlation between MESNA or NAC infusion and tHcys concentration was observed. All these facts led us to believe that LMM‐SHs, which are distinct from Hcys, are crucial for the regulation of circulating tHcys. This can be explained biochemically: plasma homocysteine is mainly present as a mixed disulfide with albumin (about 80%). This redox form has only minimal possibilities of being metabolized/eliminated. The kidney filters and reabsorbs most of the free Hcys through both low K_M_/high affinity and high K_M_/low affinity transporters [66]. The homocysteine fraction bound to albumin is not filtered but can be converted to fHcys (either thiol or disulfide) by thiol‐disulfide exchange reactions with other LMM‐SH. This is facilitated by an increase in the ratio of LMM‐SH/LMM‐SS, which is determined by GSH export (Figure 7). Higher concentrations of the free forms of homocysteine may saturate the renal transporters, considering that other amino acids (cystine, arginine, ornithine, lysine) also share the uptake system with low K_M_ and high affinity. This is supported by a clinical study in which healthy volunteers received MESNA, which resulted in a significant decrease in plasma tHcys levels and an increase in urinary free Hcys excretion [67].

In conclusion, the present study provides a rationale for considering plasma tHcys as a biomarker of cardiovascular risk rather than ascribing a direct causal role to it. We hypothesize that plasma homocysteine levels are influenced by the concentration of physiological LMM‐SH. When plasma thiol levels are elevated, mainly due to high GSH efflux, plasma tHcys concentration may be reduced by cellular uptake, metabolism and renal excretion (Figure 7). Conversely, a decrease in LMM‐SH in plasma can hinder the excretion of tHcys removal. The change of plasma thiol levels and a decrease in GSH redox potential have been associated with cardiovascular disease. For example, it has been observed that the GSH/GSSG ratio in plasma correlates with the intima‐media thickness (IMT) of the carotid artery in healthy subjects [68]. Changes in plasma tHcys levels may simply be an event triggered by the redox state of the plasma thiols. The ratio between LMM‐SH and LMM‐SS is mainly altered by GSH influx from tissues, with oxidative stress playing at best a minor role. We believe that the most important points to investigate in the near future are both the regulatory mechanisms by which GSH is released from cells and whether this phenomenon, rather than tHCys, may play a causal role in cardiovascular disease.

This study has some limitations that should be taken into account. First, it is limited to individuals who are not affected by hyperhomocysteinemia, so its results may not be generalizable to populations with elevated homocysteine levels. Second, the study is primarily biochemical in nature and does not allow for direct conclusions or hypotheses regarding the clinical impact of hyperhomocysteinemia on cardiovascular disease. Further studies, especially large‐scale clinical trials, are needed to clarify the causal relationships and the possible pathophysiological mechanisms.

Nonetheless, the mechanism and underlying reasons by which cells release significant amounts of glutathione into the extracellular space remain intriguing and require further investigation, particularly with regard to their potential role in modulating thiol homeostasis in plasma.

## Author Contributions

D.G.: data curation, investigation, writing‐original draft; S.C.: formal analysis, methodology; I.D.‐D., supervision; R.R.: conceptualization, writing‐review and editing.

## Conflicts of Interest

The authors declare no conflicts of interest.

## Data Availability

Included in article. The data that support the findings of this study are available in the Materials and Methods and Results of this article.

## References

[1] S. H. Mudd , H. L. Levy , and F. Skovby , “Disorders of Transsulfuration,” in The Metabolic and Molecular Basis of Inherited Disease, ed. C. R. Scriver , A. L. Beadet , W. S. Sly , and D. Vallee (McGraw‐Hill Publishing, 1995), 1279–1327.

[2] A. D'Angelo and J. Selhub , “Homocysteine and Thrombotic Disease,” Blood 90 (1997): 1–11. 9207431

[3] K. S. McCully , “Vascular Pathology of Homocysteinemia: Implications for the Pathogenesis of Arteriosclerosis,” American Journal of Pathology 56 (1969): 111–128. 5792556 PMC2013581

[4] D. E. L. Wilcken and B. Wilcken , “Historical Overview and Recent Perspectives,” in Homocysteine in Health and Disease, ed. R. Carmel and D. W. Jacobsen (Cambridge University Press, 2001), 1–6.

[5] G. Garibotto , A. Sofia , A. Valli , et al., “Causes of Hyperhomocysteinemia in Patients With Chronic Kidney Diseases,” Seminars in Nephrology 26 (2006): 3–7, 10.1016/j.semnephrol.2005.06.002. 16412817

[6] H. Refsum , A. D. Smith , P. M. Ueland , et al., “Facts and Recommendations About Total Homocysteine Determinations: An Expert Opinion,” Clinical Chemistry 50 (2004): 3–32, 10.1373/clinchem.2003.021634. 14709635

[7] L. L. Humphrey , R. Fu , K. Rogers , M. Freeman , and M. Helfand , “Homocysteine Level and Coronary Heart Disease Incidence: A Systematic Review and Meta‐Analysis,” Mayo Clinic Proceedings 83 (2008): 1203–1212, 10.4065/83.11.1203. 18990318

[8] D. S. Wald , M. Law , and J. K. Morris , “Homocysteine and Cardiovascular Disease: Evidence on Causality From a Meta‐Analysis,” BMJ (Clinical Research Ed.) 325 (2002): 1202, 10.1136/bmj.325.7374.1202. PMC135491 12446535

[9] M. Holmen , A. M. Hvas , and J. F. H. Arendt , “Hyperhomocysteinemia and Ischemic Stroke: A Potential Dose‐Response Association‐A Systematic Review and Meta‐Analysis,” TH Open 5 (2021): e420–e437, 10.1055/s-0041-1735978. 34595387 PMC8463136

[10] A. B. Lawrence de Koning , G. H. Werstuck , J. Zhou , and R. C. Austin , “Hyperhomocysteinemia and Its Role in the Development of Atherosclerosis,” Clinical Biochemistry 36 (2003): 431–441, 10.1016/s0009-9120(03)00062-6. 12951169

[11] P. Ganguly and S. F. Alam , “Role of Homocysteine in the Development of Cardiovascular Disease,” Nutrition Journal 14 (2015): 6, 10.1186/1475-2891-14-6. 25577237 PMC4326479

[12] K. S. McCully , “Homocysteine and the Pathogenesis of Atherosclerosis,” Expert Review of Clinical Pharmacology 8 (2015): 211–219, 10.1586/17512433.2015.1010516. 25653125

[13] S. Pushpakumar , S. Kundu , and U. Sen , “Endothelial Dysfunction: The Link Between Homocysteine and Hydrogen Sulfide,” Current Medicinal Chemistry 21 (2014): 3662–3672, 10.2174/0929867321666140706142335. 25005183 PMC5539954

[14] R. Poddar , H. Sivasubramanian , P. M. Dibello , K. Robinson , and D. Jacobsen , “Homocysteine Induces Expression and Secretion of Monocyte Chemoattractant Protein‐1 and Intereleukin‐8 in Human Aortic Endothelial Cells: Implications for Vascular Disease,” Circulation 103 (2001): 2717–2723, 10.1161/01.cir.103.22.2717. 11390343

[15] K. H. Bønaa , I. Njølstad , P. M. Ueland , et al., “Homocysteine Lowering and Cardiovascular Events After Acute Myocardial Infarction,” New England Journal of Medicine 354 (2006): 1578–1588, 10.1056/NEJMoa055227. 16531614

[16] E. Lonn , S. Yusuf , M. J. Arnold , et al., “Homocystein Lowering With Folic Acid and B Vitamins in Vascular Disease,” New England Journal of Medicine 354 (2006): 1567–1577, 10.1056/NEJMoa060900. 16531613

[17] B. A. Maron and J. Loscalzo , “The Treatment of Hyperhomocysteinemia,” Annual Review of Medicine 60 (2009): 39–54, 10.1146/annurev.med.60.041807. PMC2716415 18729731

[18] B. Cybulska and L. Kłosiewicz‐Latoszek , “Homocysteine–Is It Still an Important Risk Factor for Cardiovascular Disease?,” Kardiologia Polska 73 (2015): 1092–1096, 10.5603/KP.2015.0229. 26726726

[19] J. Li , S. Jiang , Y. Zhang , et al., “H‐Type Hypertension and Risk of Stroke in Chinese Adults: A Prospective, Nested Case‐Control Study,” Journal of Translational Internal Medicine 3 (2015): 171–178, 10.1515/jtim-2015-0027. 27847909 PMC4936453

[20] A. J. Martí‐Carvajal , I. Solà , D. Lathyris , and M. Dayer , “Homocysteine‐Lowering Interventions for Preventing Cardiovascular Events,” Cochrane Database of Systematic Reviews 8 (2017): CD006612, 10.1002/14651858.CD006612.pub5. 28816346 PMC6483699

[21] D. Giustarini , F. Galvagni , M. Orlandini , P. Fanti , and R. Rossi , “Immediate Stabilization of Human Blood for Delayed Quantification of Endogenous Thiols and Disulfides,” Journal of Chromatography. B, Analytical Technologies in the Biomedical and Life Sciences 1019 (2016): 51–58, 10.1016/j.jchromb.2016.02.009. 26896310 PMC4829439

[22] A. Vijay , L. Mohan , M. A. Taylor , et al., “The Evaluation and Use of a Food Frequency Questionnaire Among the Population in Trivandrum, South Kerala, India,” Nutrients 12 (2020): 383, 10.3390/nu12020383. 32024020 PMC7071154

[23] S. Heymsfield , C. McManus , J. Smith , V. Stevens , and D. W. Nixon , “Anthropometric Measurement of Muscle Mass: Revised Equations for Calculating Bone‐Free Arm Muscle Area,” American Journal of Clinical Nutrition 36, no. 4 (1982): 680–690, 10.1093/ajcn/36.4.680. 7124671

[24] D. Giustarini , I. Dalle‐Donne , A. Milzani , D. Braconi , A. Santucci , and R. Rossi , “Membrane Skeletal Protein S‐Glutathionylation in Human Red Blood Cells as Index of Oxidative Stress,” Chemical Research in Toxicology 32 (2019): 1096–1102, 10.1021/acs.chemrestox.8b00408. 30945548

[25] Z. Tao , R. A. Raffel , A. K. Souid , and J. Goodisman , “Kinetic Studies on Enzyme‐Catalyzed Reactions: Oxidation of Glucose, Decomposition of Hydrogen Peroxide and Their Combination,” Biophysical Journal 96 (2009): 2977–2988, 10.1016/j.bpj.2008.11.071. 19348778 PMC2711289

[26] D. Giustarini , I. Dalle‐Donne , R. Colombo , et al., “Protein Glutathionylation in Erythrocytes,” Clinical Chemistry 49 (2003): 327–330, 10.1373/49.2.327. 12560364

[27] G. Ellman and H. Lysko , “A Precise Method for the Determination of Whole Blood and Plasma Sulfhydryl Groups,” Analytical Biochemistry 93 (1979): 98–102, 10.1016/S0003-2697(79)80122-0. 434474

[28] D. Giustarini , I. Dalle‐Donne , S. Lorenzini , et al., “Protein Thiolation Index (PTI) as a Biomarker of Oxidative Stress,” Free Radical Biology & Medicine 53 (2012): 907–915, 10.1016/j.freeradbiomed.2012.06.022. 22732185

[29] R. E. Hansen , D. Roth , and J. R. Winther , “Quantifying the Global Cellular Thiol‐Disulfide Status,” Proceedings of the National Academy of Sciences of the United States of America 106 (2009): 422–427, 10.1073/pnas.0812149106. 19122143 PMC2626718

[30] G. Colombo , M. Clerici , M. E. Garavaglia , et al., “A Step‐by‐Step Protocol for Assaying Protein Carbonylation in Biological Samples,” Journal of Chromatography. B, Analytical Technologies in the Biomedical and Life Sciences 1019 (2016): 178–190, 10.1016/j.jchromb.2015.11.052. 26706659

[31] D. Giustarini , P. Del Soldato , A. Sparatore , and R. Rossi , “Modulation of Thiol Homeostasis Induced by H2S‐Releasing Aspirin,” Free Radical Biology & Medicine 48 (2010): 1263–1272, 10.1016/j.freeradbiomed.2010.02.014. 20171274

[32] M. M. Bradford , “A Rapid and Sensitive Method for the Quantitation of Microgram Quantities of Protein Utilizing the Principle of Protein‐Dye Binding,” Analytical Biochemistry 72 (1976): 248–254, 10.1006/abio.1976.9999. 942051

[33] E. E. Di Iorio , “[4] Preparation of Derivatives of Ferrous and Ferric Hemoglobin,” Methods in Enzymology 76 (1981): 57–72, 10.1016/0076-6879(81)76114-7. 7329277

[34] S. E. Moriarty‐Craige and D. P. Jones , “Extracellular Thiols and Thiol/Disulfide Redox in Metabolism,” Annual Review of Nutrition 24 (2004): 481–509. 10.1146/annurev.nutr.24.012003.132208 15189129

[35] O. O. Abugo and J. M. Rifkind , “Oxidation of Hemoglobin and the Enhancement Produced by Nitroblue Tetrazolium,” Journal of Biological Chemistry 269 (1994): 24845–24853. 7929164

[36] D. Giustarini , P. Fanti , A. Sparatore , E. Matteucci , and R. Rossi , “Anethole Dithiolethione Lowers the Homocysteine and Raises the Glutathione Levels in Solid Tissues and Plasma of Rats: A Novel Non‐Vitamin Homocysteine‐Lowering Agent,” Biochemical Pharmacology 89 (2014): 246–254, 10.1016/j.bcp.2014.03.005. 24637238 PMC4142420

[37] D. Giustarini , I. Dalle‐Donne , S. Lorenzini , A. Milzani , and R. Rossi , “Age‐Related Influence on Thiol, Disulfide, and Protein‐Mixed Disulfide Levels in Human Plasma,” Journals of Gerontology. Series A, Biological Sciences and Medical Sciences 61 (2006): 1030–1038, 10.1093/gerona/61.10.1030. 17077195

[38] D. Giustarini , I. Dalle‐Donne , A. Milzani , and R. Rossi , “Low Molecular Mass Thiols, Disulfides and Protein Mixed Disulfides in Rat Tissues: Influence of Sample Manipulation, Oxidative Stress and Ageing,” Mechanisms of Ageing and Development 132 (2011): 141–148, 10.1016/j.mad.2011.02.001. 21335026

[39] R. Rossi , D. Giustarini , S. Fineschi , G. De Cunto , G. Lungarella , and E. Cavarra , “Differential Thiol Status in Blood of Different Mouse Strains Exposed to Cigarette Smoke,” Free Radical Research 43 (2009): 538–545, 10.1080/10715760902893332. 19370473

[40] D. Giustarini , I. Dalle‐Donne , E. Cavarra , et al., “Metabolism of Oxidants by Blood From Different Mouse Strains,” Biochemical Pharmacology 71 (2006): 1753–1764, 10.1016/j.bcp.2006.03.015. 16624256

[41] V. Bocci , N. Di Paolo , G. Garosi , et al., “Ozonation of Blood During Extracorporeal Circulation. I. Rationale, Methodology and Preliminary Studies,” International Journal of Artificial Organs 22 (1999): 645–651. 10532435

[42] W. Dröge , “Aging‐Related Changes in the Thiol/Disulfide Redox State: Implications for the Use of Thiol Antioxidants,” Experimental Gerontology 37 (2002): 1333–1345, 10.1016/S0531-5565(02)00175-4. 12559403

[43] D. A. Keire , E. Strauss , W. Guo , B. Noszál , and D. L. Rabenstein , “Kinetics and Equilibria of Thiol/Disulfide Interchange Reactions of Selected Biological Thiols and Related Molecules With Oxidized Glutathione,” Journal of Organic Chemistry 57 (1992): 123–127, 10.1021/jo00027a023.

[44] L. Turell , R. Radi , and B. Alvarez , “The Thiol Pool in Human Plasma: The Central Contribution of Albumin to Redox Processes,” Free Radical Biology & Medicine 65 (2013): 244–253, 10.1016/j.freeradbiomed.2013.05.050. 23747983 PMC3909715

[45] S. Sengupta , C. Wehbe , A. K. Majors , M. E. Ketterer , P. M. DiBello , and D. W. Jacobsen , “Relative Roles of Albumin and Ceruloplasmin in the Formation of Homocystine, Homocysteine‐Cysteine‐Mixed Disulfide, and Cystine in Circulation,” Journal of Biological Chemistry 276 (2001): 46896–46904, 10.1074/jbc.M108451200. 11592966

[46] O. W. Griffith , “Biologic and Pharmacologic Regulation of Mammalian Glutathione Synthesis,” Free Radical Biology & Medicine 27 (1999): 922–935, 10.1016/s0891-5849(99)00176-8. 10569625

[47] M. H. Stipanuk , J. E. Dominy, Jr. , J. I. Lee , and R. M. Coloso , “Mammalian Cysteine Metabolism: New Insights Into Regulation of Cysteine Metabolism,” Journal of Nutrition 136 (2006): 1652S–1659S, 10.1093/jn/136.6.1652S. 16702335

[48] S. Bannai , “Transport of Cystine and Cysteine in Mammalian Cells,” Biochimica et Biophysica Acta 779 (1984): 289–306, 10.1016/0304-4157(84)90014-5. 6383474

[49] R. G. Knickelbein , T. Seres , G. Lam , R. B. Johnston, Jr. , and J. B. Warshaw , “Characterization of Multiple Cysteine and Cystine Transporters in Rat Alveolar Type II Cells,” American Journal of Physiology 273 (1997): L1147–L1155, 10.1152/ajplung.1997.273.6.L1147. 9435569

[50] M. Orlowski and A. Meister , “The Gamma‐Glutamyl Cycle: A Possible Transport System for Amino Acids,” Proceedings of the National Academy of Sciences of the United States of America 67 (1970): 1248–1255, 10.1073/pnas.67.3.1248. 5274454 PMC283344

[51] R. G. Deeley , C. Westlake , and S. P. Cole , “Transmembrane Transport of Endo‐ and Xenobiotics by Mammalian ATP‐Binding Cassette Multidrug Resistance Proteins,” Physiological Reviews 86 (2006): 849–899, 10.1152/physrev.00035.2005. 16816140

[52] N. Ballatori , S. M. Krance , R. Marchan , and C. L. Hammond , “Plasma Membrane Glutathione Transporters and Their Roles in Cell Physiology and Pathophysiology,” Molecular Aspects of Medicine 30 (2009): 13–28, 10.1016/j.mam.2008.08.004. 18786560 PMC2716123

[53] H. Vázquez‐Meza , M. M. Vilchis‐Landero , M. Vázquez‐Carrada , D. Uribe‐Ramírez , and D. Matuz‐Mares , “Cellular Compartmentalization, Glutathione Transport and Its Relevance in Some Pathologies,” Antioxidants 12 (2023): 834, 10.3390/antiox12040834. 37107209 PMC10135322

[54] M. Ookhtens and N. Kaplowitz , “Role of the Liver in Interorgan Homeostasis of Glutathione and Cyst(e)ine,” Seminars in Liver Disease 18 (1998): 313–329, 10.1055/s-2007-1007167. 9875551

[55] D. Giustarini , A. Milzani , I. Dalle‐Donne , and R. Rossi , “Red Blood Cells as a Physiological Source of Glutathione for Extracellular Fluids,” Blood Cells, Molecules & Diseases 40 (2008): 174–179, 10.1016/j.bcmd.2007.09.001. 17964197

[56] B. Büdy , R. O'Neill , P. M. DiBello , S. Sengupta , and D. W. Jacobsen , “Homocysteine Transport by Human Aortic Endothelial Cells: Identification and Properties of Import Systems,” Archives of Biochemistry and Biophysics 446 (2006): 119–130, 10.1016/j.abb.2005.12.014. 16455044 PMC2846170

[57] B. Hultberg , A. Andersson , and A. Isaksson , “Higher Export Rate of Homocysteine in a Human Endothelial Cell Line Than in Other Human Cell Lines,” Biochimica et Biophysica Acta 1448, no. 1 (1998): 61–69, 10.1016/s0167-4889(98)00119-0. 9824669

[58] G. Garibotto , A. Sofia , S. Saffioti , et al., “Interorgan Exchange of Aminothiols in Humans,” American Journal of Physiology. Endocrinology and Metabolism 284 (2003): E757–E763, 10.1152/ajpendo.00403.2002. 12475755

[59] W. Herrmann and M. Herrmann , “The Controversial Role of HCY and Vitamin B Deficiency in Cardiovascular Diseases,” Nutrients 14 (2022): 1412, 10.3390/nu14071412. 35406025 PMC9003430

[60] Homocysteine Studies Collaboration , “Homocysteine and Risk of Ischemic Heart Disease and Stroke: A Meta‐Analysis,” Journal of the American Medical Association 288, no. 16 (2002): 2015–2022, 10.1001/jama.288.16.2015. 12387654

[61] J. Loscalzo , “Homocysteine Trials–Clear Outcomes for Complex Reasons,” New England Journal of Medicine 354 (2006): 1629–1632, 10.1056/NEJMe068060. 16531615

[62] S. Kaul , A. A. Zadeh , and P. K. Shah , “Homocysteine Hypothesis for Atherothrombotic Cardiovascular Disease: Not Validated,” Journal of the American College of Cardiology 48 (2006): 914–923, 10.1016/j.jacc.2006.04.086. 16949480

[63] P. Ventura , R. Panini , M. C. Pasini , G. Scarpetta , and G. Salvioli , “N‐Acetyl‐Cysteine Reduces Homocysteine Plasma Levels After Single Intravenous Administration by Increasing Thiols Urinary Excretion,” Pharmacological Research 40 (1999): 345–350, 10.1006/phrs.1999.0519. 10527647

[64] N. Shukla , A. Koupparis , R. A. Jones , G. D. Angelini , R. Persad , and J. Y. Jeremy , “Penicillamine Administration Reverses the Inhibitory Effect of Hyperhomocysteinaemia on Endothelium‐Dependent Relaxation and Superoxide Formation in the Aorta of the Rabbit,” European Journal of Pharmacology 531 (2006): 201–208, 10.1016/j.ejphar.2005.12.003. 16451799

[65] B. H. Lauterburg , T. Nguyen , B. Hartmann , E. Junker , A. Küpfer , and T. Cerny , “Depletion of Total Cysteine, Glutathione, and Homocysteine in Plasma by Ifosfamide/Mesna Therapy,” Cancer Chemotherapy and Pharmacology 35 (1994): 132–136, 10.1007/BF00686635. 7987989

[66] J. W. Foreman , H. Wald , G. Blumberg , L. M. Pepe , and S. Segal , “Homocystine Uptake in Isolated Rat Renal Cortical Tubules,” Metabolism 31 (1982): 613–619, 10.1016/0026-0495(82)90101-9. 6804755

[67] B. L. Urquhart , D. J. Freeman , J. D. Spence , and A. A. House , “The Effect of Mesna on Plasma Total Homocysteine Concentration in Hemodialysis Patients,” American Journal of Kidney Diseases 49 (2007): 109–117, 10.1053/j.ajkd.2006.10.002. 17185151

[68] S. Ashfaq , J. L. Abramson , D. P. Jones , et al., “The Relationship Between Plasma Levels of Oxidized and Reduced Thiols and Early Atherosclerosis in Healthy Adults,” Journal of the American College of Cardiology 47 (2006): 1005–1011, 10.1016/j.jacc.2005.09.063. 16516085

